# The Enduring Effects of Parental Alcohol, Tobacco, and Drug Use on Child Well-being: A Multilevel Meta-Analysis

**DOI:** 10.1017/S0954579419000749

**Published:** 2020-05

**Authors:** Sofie Kuppens, Simon C. Moore, Vanessa Gross, Emily Lowthian, Andy P. Siddaway

**Affiliations:** 1 Department of Public Health and Primary Care, KU Leuven, Centre for Environment and Health, Leuven, Belgium; 2 Karel de Grote University of Applied Sciences and Arts, Antwerp, Belgium; 3 School of Dentistry, Cardiff University, Cardiff, Wales, UK; 4 Crime and Security Research Institute Friary House, Cardiff, Wales, UK; 5 DECIPHer, Cardiff School of Social Sciences, Cardiff University, Cardiff, Wales, UK; 6 Stirling Management School, University of Stirling, Stirling, Stirlingshire, Scotland, UK

**Keywords:** alcohol, children, meta-analysis, parent, substance abuse, tobacco, well-being

## Abstract

The effects of psychoactive substance abuse are not limited to the user, but extend to the entire family system, with children of substance abusers being particularly at risk. This meta-analysis attempted to quantify the longitudinal relationship between parental alcohol, tobacco, and drug use and child well-being, investigating variation across a range of substance and well-being indices and other potential moderators. We performed a literature search of peer-reviewed, English language, longitudinal observational studies that reported outcomes for children aged 0 to 18 years. In total, 56 studies, yielding 220 dependent effect sizes, met inclusion criteria. A multilevel random-effects model revealed a statistically significant, small detriment to child well-being for parental substance abuse over time (*r* = .15). Moderator analyses demonstrated that the effect was more pronounced for parental drug use (*r* = .25), compared with alcohol use (*r* = .13), tobacco use (*r* = .13), and alcohol use disorder (*r* = .14). Results highlight a need for future studies that better capture the effect of parental psychoactive substance abuse on the full breadth of childhood well-being outcomes and to integrate substance abuse into models that specify the precise conditions under which parental behavior determines child well-being.

*Registration*: PROSPERO CRD42017076088

The effects of psychoactive substance abuse are not limited to the user but extend to the entire family system and society at large. In line with the growing body of research on the detrimental influence of adverse childhood experiences, children of users are particularly at risk, with effects on their health, well-being, and their own use of tobacco, alcohol, or drugs (e.g., National Center on Addiction and Substance Abuse, 2005). Millions of children are likely to be affected by parental psychoactive substance abuse, as estimates suggest that 12.3% of US children aged 17 years or younger reside in a home with at least one parent with a substance abuse disorder (Lipari & Van Horn, 2017). In Europe, the estimates for children under the age of 20 years living with alcohol abusing parents varies greatly and ranges from 5.7% in Finland, 10.5% in Denmark, 15.4% in Germany, and 17% to 23% in Poland (European Monitoring Centre for Drugs and Drug Addiction, 2008).

A large body of evidence spanning several decades has documented that the children of parents who abuse alcohol, tobacco, and drugs (henceforth collectively referred to as parental substance abuse) are more likely to develop a variety of emotional, behavioral, physical, cognitive, academic, and social problems in the short and long run (e.g., Barnard & McKeganey, 2004; Straussner & Fewell, 2011). For example, passive tobacco exposure has been linked to somatic health problems in children and adolescents, together with an increased risk of children's own tobacco use initiation and dependence (Hussong et al., 2008). In addition, parental substance abuse has been linked to family breakdown, which is a key risk factor for children's poor mental health (Mallett, Rosenthal, & Keys, 2005; Størksen, Røysamb, Moum, & Tambs, 2005). It has also been linked to a reduction in the quality of the parent-child relationship and maladaptive relationship models that can be detrimental to the development of later peer relationships (Fearon, Bakermans, Kranenburg, Van Ijzendoorn, Lapsley, & Roisman, 2010; Hoeve et al., 2012).

Parental substance abuse has also been associated with a reduction in the extent that parents monitor their children, which may undermine parents’ ability to provide a safe and nurturing home environment (Barnard & McKeganey, 2004). Instability with respect to employment, family structure, housing, childcare, and household finances has also been shown to co-occur with parental substance abuse, with consequences that extend beyond the family environment to influence children's social functioning (Berger, Paxson, & Waldfogel, 2009; De Goede, Branje, Delsing, & Meeus, 2009; Giesbrecht, Cukier, & Steeves, 2010; Lander, Howsare, & Byrne, 2013; Martin, Razza, & Brooks-Gunn, 2012; Öberg, Jaakkola, Woodward, Peruga, & Prüss-Ustün, 2011; Parsons, Adler, & Kaczala, 1982).

There appears to be a general consensus amongst researchers, clinicians, and policy makers that parental substance abuse negatively affects child well-being (Bountress & Chassin, 2015; Hussong, Huang, Curran, Chassin, & Zucker, 2010; McGrath, Watson, & Chassin, 1999; Peterson et al., 2006; Puttler, Zucker, Fitzgerald, & Bingham, 1998; Rossow, Keating, Felix, & McCambridge, 2016). However, the extent and nature of this relationship is currently unknown, because findings in the literature vary considerably in magnitude and studies have typically focused on a subset of child well-being outcomes or a specific parental substance abuse type. Furthermore, there is considerable variation in the way that parental substance abuse and child well-being outcomes have each been operationalized. A systematic synthesis of the available evidence using an overarching framework is needed to quantify the extent to which parental substance abuse predicts detrimental child well-being outcomes over time in order to draw more general conclusions and to determine the degree of heterogeneity in this relationship while also identifying factors that could explain any inconsistencies. In doing so, it would also be possible to identify gaps in the research literature and key directions for future research.

## The present meta-analysis

To address these important knowledge gaps, we conducted a systematic review and meta-analysis of the longitudinal relationship between parental psychoactive substance abuse and child well-being that included multiple substance abuse types and well-being domains. We focused on longitudinal studies, as this provides insight into the directionality of any effect and the long-term characteristics of any association. We used a state-of-the-art multilevel meta-analytic approach that allowed us to model the dependency among effect sizes, which is common in primary studies, in part because they include multiple outcome measures and multiple informants, or they report on multiple family members. Given the broad and comprehensive nature of this meta-analysis, heterogeneity within the results was expected. Therefore, the following study and sample characteristics that could potentially moderate the strength of the relationship between substance abuse and well-being were examined.

Parental substance abuse refers to the consumption of psychoactive substances, including licit and illicit substances, of which alcohol (Rossow et al., 2016) and tobacco (Saulyte, Regueira, Montes-Martínez, Khudyakov, & Takkouche, 2014) are the most frequently used. Given differences in the legal and social status, addictive potential, and cognitive effects, the effect on family members has been proposed to vary according to the particular substance consumed (Straussner, 1994; Straussner & Fewell, 2011). Apart from the different types, the use of such substances have commonly been considered to range on a continuum from recreational use to chronic dependence (Straussner, 2004). Substance dependence may vary in severity from mild to severe and refers to compulsive and continued use irrespective of any adverse consequences. We hypothesised that the association with child well-being would be less pronounced for the use of licit substances of a recreational nature.

Well-being is a nebulous term that is informed by personal, cultural, and other factors (Diener, Oishi, & Lucas, 2003), and there is no clear distinction between well-being and quality of life or mental health problems (Siddaway, Taylor, & Wood, 2018; Siddaway, Wood, & Taylor, 2017). Well-being is typically considered a multidimensional construct (Gallagher, Lopez, & Preacher, 2009; Keyes, 2009; Ryff & Keyes, 1995) that includes both subjective and objective features (Newman, Tay, & Diener, 2014) and, for young people, can refer to domains as disparate as relationships, health, activities, finance, education, and skills (Bradshaw, 2016). Because existing research has largely focused on a specific well-being domain, a comprehensive examination of whether and how well-being subtype moderates the substance abuse–well-being relationship is currently lacking. We sought to address this gap by conceptualizing child well-being as a broad, multidimensional construct that involves physical, psychological, cognitive, social, and economic subdomains. This broad conceptualization is similar to the World Health Organization's (WHO, 2004, p. 10) definition of mental and physical health (“a state of complete physical, mental and social well-being”).

Although parental substance abuse can be detrimental at any point in a child's life, it is feasible that its effects could vary according to the age of the child. For example, parental tobacco use in a child's first year of life has been associated with a greater risk of physical health symptoms (Mitchell et al., 2012). For substance abuse, it has been argued that, compared with younger children, adolescents are at greater risk due to more prolonged exposure to parental substance abuse (Straussner & Fewell, 2011). We therefore examined whether effects differed according to the age of the child.

Although substance abuse by the mother and the father are both important, previous research has often found more pronounced associations with multiple adverse child outcomes for maternal substance abuse (Mitchell et al., 2012; Straussner & Fewell, 2011). These gender differences may be due to children spending more time in the presence of their mothers than their fathers, as mothers traditionally take a more active role in child rearing. It is also possible that parental substance abuse effects may manifest differently in boys and girls. For example, associations between parental smoke exposure and child mental health outcomes have been found to be more apparent for boys (Bandiera, Richardson, Lee, He, & Merikangas, 2011). Given previous findings on gender differences, we examined whether the gender of parent and child moderates the substance abuse–well-being relationship.

The choice of informants in behavioral research has been a subject of a long-lasting debate. For many measures of child functioning and parenting behavior, inter-informant agreement is low-to-moderate because parent and child reports are often discrepant (De Los Reyes et al., 2015; Kuppens, Grietens, Onghena, & Michiels, 2009b). Similarly, for licit and illicit substances, there is variation in prevalence by data collection mode (Beck, Guignard, & Legleye, 2014). Such inconsistent research findings may reflect method bias related to the specific informant or data collection mode (e.g., socially desirable responding, shared method variance; Kuppens, Grietens, Onghena, & Michiels, 2009a; Podsakoff, MacKenzie, Lee, & Podsakoff, 2003; |Tourangeau & Yan, 2007). As the potential method effect remains unexplored for the parental substance abuse–child well-being relationship, we examined the moderating role of informant and data collection mode.

Finally, the interval adopted in longitudinal studies can vary considerably, which in turn may influence the strength of the relationship between substance abuse and well-being over time for several reasons (Collins & Horn, 1991). The strength of the relationship could potentially decrease over time, as there is increasing opportunity for other cognitive, biological, and environmental variables to exert an effect. In addition, respondents may more easily remember their answers to a previous assessment when the time interval is shorter. We hypothesized that longitudinal relationships would be less pronounced as the time interval between parental substance abuse exposure and assessment of child well-being outcomes increased.

## Method

This meta-analysis was conducted in accordance with guidelines for conducting a systematic review (Siddaway, Wood, & Hedges, 2019), the Preferred Reporting Items for Systematic Reviews and Meta-analyses (Moher, Liberati, Tetzlaff, Altman, & The PRISMA Group, 2009) and Meta-Analysis of Observational Studies in Epidemiology (Stroup et al., 2000) standards.

### Literature search

Four electronic databases (PubMed, Medline, Embase, PsychInfo) were searched from inception to June 26, 2017. In addition, reference lists from eligible publications and relevant reviews were hand-searched. Comprehensive search strategies were developed by combining key and index terms covering the concepts of *parental substance abuse* AND *child well-being* (or related terms, see Supplement).

### Inclusion and exclusion criteria

Peer-reviewed, English language, longitudinal observational studies (Morrison et al., 2012) were eligible for inclusion. Studies were required to include at least one association over time between a measure of parental substance abuse (measured at Time 1) and well-being (measured at Time 2) for children aged 18 years or younger. As previous research revealed polysubstance abuse in parental substance abuse (Hussong et al., 2008; Straussner & Fewell, 2011), we included recreational and disordered (including clinically defined dependence) alcohol, tobacco, and drug use. We included studies reporting comorbid substance use, as this is frequently observed in dependent users and excluding such groups might bias results towards being more conservative and less generalizable to the general population.

We operationalized child well-being broadly according to five distinct domains, in-keeping with a narrative literature review of how well-being can be conceptualized (Pollard & Lee, 2003), and in order to achieve a comprehensive overview of subjective and objective well-being of children, namely, (a) physical (including overall health and risks to health); (b) psychological (including emotional and mental states of mind); (c) cognitive (including capacity for learning and recall, academic achievement, and learning disability); (d) social (including social relationships and behaviors and anti-social behavior); and (e) economic (whether outside financial support is required) well-being. As we attempted to provide estimates of the association in the general population, studies were excluded if they were sampled from specific groups experiencing conditions that were likely to significantly affect associations between parental substance abuse and child well-being (e.g., detained adolescents, parents diagnosed with HIV). One author (V.G.) screened titles and abstracts, and S.M. rechecked extraction methods. Two authors (V.G. and S.M.) screened full text articles for inclusion, *N* = 381, kappa = .93, 95% CI [.92, .99]. References were exported and managed with Endnote X7.

### Coding

To capture as much variation in substance abuse type, effect sizes were categorized into “Alcohol,” “Alcohol Use Disorder,” “Drugs,” “Drug Use Disorder,” “Tobacco,” and a non-descript category, “Any substance.” We used “Use Disorder” to identify parents whose use of substances was clinically significant and recorded using a clinically validated instrument. Child well-being was coded according to the aforementioned five domains, namely physical, psychological, social, cognitive, or economic well-being.

Several other study and outcome characteristics were extracted from each study: year; country from which the sample was drawn; sample size; retention rate; sample type (general population or specific subpopulation such as school children); parent and child gender, child age (0–5, 6–11, or 12–18 years); data collection mode for child and parent variables (interview, questionnaire); measurement instrument (standardized diagnostic, validated but nondiagnostic, or unstandardized); informant (parent, child, or teacher report); and follow-up duration (number of data collection points and the time in months between first and last assessment).

### Data analysis

#### Effect size calculations

Pearson correlations (*r*) were computed to represent associations such that a positive correlation denoted that higher levels of parental substance abuse were associated with lower child well-being over time. Results reported in another metric were transformed to *r* whenever possible (Wilson, 2017). Study authors were contacted for additional information when an effect size or data to construct an effect size were not reported. Before pooling effect sizes, the correlations were transformed using Fisher's *Z*_*r*_ transformation (Rosenthal, 1991). Pooled *Z*_r_ expressions were transformed back to *r* expressions for reporting.

#### Meta-analytic integration

As most studies (71%) reported more than one relevant effect size, the independence assumption that underlies traditional meta-analysis was violated. Common approaches to addressing such dependency include choosing only one effect size from many or averaging across effect sizes within studies. Although such approaches allow one to adopt traditional meta-analytic techniques, they also result in a loss of information, rule out critical analyses of within-study moderators (e.g., well-being outcome differences), and may distort meta-analytic results (e.g., Cheung & Chan, 2004). We therefore employed a multilevel random-effects meta-analysis that permitted incorporating all of the relevant effect sizes from each study, while accounting for dependency among these effect sizes with SAS PROC HPMIXED (Littell, Milliken, Stroup, Wolfinger, & Schabenberger, 2006; Van den Noortgate, Lopez-Lopez, Marin-Martinez, & Sanchez-Meca, 2013).

The meta-analysis was implemented as a three-level model that models sampling variation for each effect size (Level 1), within-study variation (Level 2), and between-study variation (Level 3) (Van den Noortgate et al., 2013). We first computed an overall estimate of the longitudinal association between parental substance abuse and child well-being in a random-effects model. Between-study heterogeneity was reflected by the between-study variance (Level 3, representing differences between studies), but the three-level model also yielded an estimate of the within-study variance (Level 2, representing differences between effect sizes within studies). A likelihood ratio test was used to test the variation in effect sizes between and within studies, while the ratio of the variance at each level and the total variance was computed to reflect the amount of variance situated between and within studies. Subsequently, six substantive (study region, child age, child gender, parent gender, parental consumption type, child well-being type) and six methodological moderators were examined with grand-mean centering applied to continuous moderators. Separate mixed-effects models were fitted for each moderator variable to avoid inflating Type II error (Raudenbush & Bryk, 2002), and subgroup analyses were only conducted when data from at least four studies were available (Fu et al., 2011).

#### Risk of bias

In the absence of a standard risk of bias tool for longitudinal observational studies (see Siddaway et al., 2019), six criteria were adopted to reflect the methodological rigor of each study (Faragher, Cass, & Cooper, 2005; Higgins & Green, 2011; Wong, Cheung, & Hart, 2008): whether the study sample size provided adequate statistical power (calculated as at least *N* = 193 to provide a power of .80 in order to detect *r* = .20 with α = .05), retention rate, representativeness of the sample, standardized assessment tool for parental substance abuse, standardized assessment tool for child well-being, and whether associations were adjusted for confounding. We tested whether effect sizes differed according to the separate criteria.

Three strategies were used to assess publication bias. First, a funnel plot was created to visually search for evidence of bias, which would be apparent in an asymmetrical plot. Next, asymmetry was assessed using Egger's weighted regression test (Egger, Smith, Schneider, & Minder, 1997; Torgerson, 2006). Third, a sensitivity analysis was performed, which applies different a priori weight functions to correct the population effect size estimate for different types and severities of potential publication bias (Vevea & Woods, 2005). A mean study effect size was used in the publication bias analyses because independence is assumed in these methods and it is not yet possible to account for effect size dependency in these tests.

## Results

### Study characteristics

**Figure 1.**
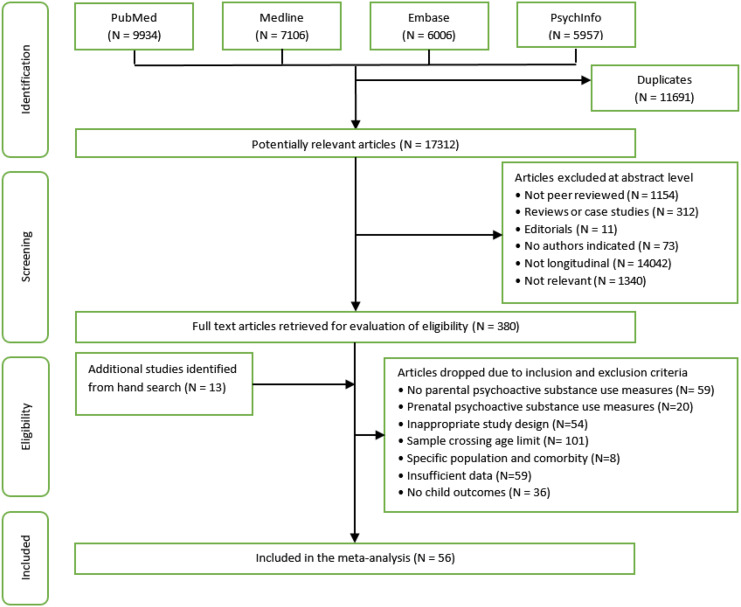
Flow diagram of study identification and selection, including reasons for exclusion.

The meta-analysis included 56 studies and 220 effect sizes. A flow diagram of study identification and selection is presented in Figure 1. All 56 studies explicitly stated or used terminology implying that children and parents cohabited. Sample sizes varied from 83 to 49,5. Most studies drew samples from a general (34%) or school (27%) population; the remaining studies sampled from clinically dependent parents (22%) or used a specific sampling frame (e.g., 16% of families living in rural areas). Most studies (*n* =35) were conducted in North America, followed by Europe (*n* = 9), Australasia (*n* = 9), Asia (*n* = 2), and South America (*n* = 1).

Drug use was variously defined and predominantly included cannabis use. Most of the effect sizes (78%) examined outcomes in young people aged 12 to 18 years and on average included 45% female children. For parental consumption, 37% and 27% of reported effect sizes referred to maternal and paternal behavior, respectively, and 36% reported on the consumption for both parents combined. Effect sizes were evenly split across alcohol (38%) and tobacco (32%) use; drug use and alcohol use disorder were assessed in 6% and 19% of the effect sizes, respectively. The remaining effect sizes (5%) considered “any substance abuse.” No studies considered prescription medication. Information on parental substance abuse was mainly retrieved directly from parents (72%) or their children (25%). Most child well-being effect sizes concerned physical (74%) or psychological (19%) well-being, while social, cognitive, and economic well-being were assessed less frequently (7%). Well-being was mainly child (76%) or parent reported (15%). Time between parental substance abuse measurement and child well-being outcome averaged 60.26 months across all effect sizes (*SD* = 47.90, range = 3–190 months). Table 1 presents descriptive and mean effects sizes for the 56 included studies.

**Table 1.** Descriptives and mean effect size for studies included in the meta-analysis (*N* = 56).

Study	Country	Child age (years)	Gender child (% female)	Gender parents	Parental consumption	Child Well-being	Informant (parental consumption)	Informant (child well-being)	Method of assessment	Time lag (months)	Mean *N*	Mean *r*	Lower 95% CI	Upper 95% CI
Adalbjarnardottir & Rafnsson, 2002	Iceland	12–18	54%	B	T, A	Tobacco, Alcohol	Child	Self-report	Q	36	928	.10	.03	.17
Barman, et al., 2004	Finland	12–18	50%	MF	T	Tobacco	Self-report	Self-report	Q	36	4739	.20	.13	.27
Barnow, et al., 2007	USA	12–18	48%	B	A	Behavioral problems	Self-report	Parent	I	60	83	.06	−.15	.26
Bor, McGee, & Fagan, 2004	Australia	12–18	48%	M	T	Antisocial behavior	Self-report	Mother	Q	168	5278	.09	−.10	.28
Brook, et al., 2001	Colombia	12–18	49%	MF	T, D	Drug use	Self-report	Self-report	I	24	1374	.12	.02	.21
Bufferd et al., 2014	USA	6–11	46%	B	DD	Depression	Self-report	Self-report	I	36	456	.15	.00	.29
Burke, et al., 2001	Australia	12–18	53%	MF	A	Alcohol	Self-report	Self-report	Q		798	.26	.15	.37
Chaffin, et al., 1996	USA	missing	43%	B	A	Physical abuse, Neglect	Self-report	Parents	I	12	7103	.30	.17	.41
Colder, et al., 1997	USA	12–18	43%	MF	AD	Heavy alcohol use	Self-report	Self-report	Q	36	428	.17	.02	.31
Connolly, et al., 1992	USA	12–18	missing	M	A	Behavior problems	Self-report	Self-report, Teacher	I	48	652	.05	−.09	.19
Crandall, et al., 2006	USA	0–5	0%	M	A	Head injury	Self-report	Mother	I	12	3808	.21	.02	.38
Cranford, et al., 2010)	USA	12–18	31%	MF	AD	Alcohol	Self-report	Self-report	I	72	148	.21	.12	.29
Dwyer, et al., 1999	Tasmania	0–5	49%	MF	T	Sudden Infant Death Syndrome	Self-report	Parent	I	3	9788	.26	.19	.32
Eiden, et al., 2007	USA	0–5	48%	B	A	Externalizing behavior	Self-report	Mother, Father, Teacher	I	48	227	.03	−.06	.13
Ellickson, et al., 2001	USA	12–18	missing	B	A	Problem Alcohol	Self-report	Self-report	Q	60	4283	.04	−.16	.22
Fergusson, et al., 1993	New Zealand	12–18	missing	M	T	Conduct disorder, Attention deficit, Total disruptive behavior	Self-report	Mother, Teacher	Q	84	960	.16	.07	.23
Fergusson, et al., 1994	New Zealand	12–18	missing	B	D	Multiple problem behaviors	Self-report	Self-report	Q	48	942	.07	−.13	.26
Fergusson, et al., 1996	New Zealand	12–18	missing	B	T	Depression, Nicotine dependence	Self-report	Self-report	Q	48	942	.10	−.04	.24
Frank et al., 2014	USA	12–18	0%	B	D	Drug use	Other	Self-report	I	180	157	.34	.11	.54
Fuller et al., 2003	USA	6–11	53%	B	AD	Aggression	Self-report	Parent	Q	36	186	.15	−.09	.37
Griesler, et al., 2011	USA	12–18	63%	B	T	Nicotine dependence	Self-report	Self-report	I	24	814	.15	−.05	.34
Gritz et al., 2003	USA	12–18	missing	B	T	Tobacco	Self-report	Self-report	Q	12	659	.11	−.10	.30
Hanewinkel et al., 2014	Germany	12–18	57%	B	A	Alcohol		Self-report	Q	12	13642	.11	−.03	.24
Heron et al., 2013	UK	12–18	51%	M	A, T, D	Problematic drug use, Non-problematic drug use	Self-report	Self-report	Q	190	4159	.25	.18	.32
Hoffmann & Cerbone, 2002	USA	12–18	Mixed/ boys only/ girls only	B	DD	Drug abuse, Alcohol abuse	Self-report	Self-report	I	96	804	.25	.11	.38
Hussong & Chassin, 1997	USA	12–18	51%	B	AD	Heavy alcohol use	Self-report	Self-report	I	36	293	.08	−.05	.20
Johnson & Leff, 1999	USA	0–5	51%	B	T	IQ	Self-report	Self-report	I	48	122	.03	−.23	.28
Kaplow, et al., 2002	USA	12–18	51%	B	D	Drug use	Self-report	Self-report	I	84	295	.24	.02	.43
Knudsen, et al., 2015	Norway	0–5	52%	M	A	Externalising & Internalising behavior problems	Self-report	Mother	Q	18	49553	.38	.26	.49
Korhonen et al., 2008	Finland	12–18	61%	MF	A, T	Drug use	Self-report	Self-report	Q	72	4138	.14	.08	.21
Kotch et al., 2014	USA	12–18	49%	B	T	Externalising behavior problems	Self-report	Self-report	I	72	915	.10	−.10	.29
Lessard et al., 2014	USA	12–18	52%	MF	T, D, AD	E-cigarette use	Self-report	Self-report	Q	48	136	.10	−.01	.21
Li, et al., 2002	USA	6–11	missing	B	A, T, D, A	Alcohol, Tobacco, Drug use, Any substance use	Self-report	Self-report	Q		1551	.14	.09	.19
Lindsey et al., 2008	USA	6–11	0%	missing	A	Externalising & Internalising behavior problems	Self-report	Self-report	I	48	514	.15	.01	.29
Loukas, et al., 2001	USA	6–11	52%	B	AD	Externalising behavior	Self-report	Parent	I	36	208	.27	.04	.47
Malone, et al., 2002	USA	12–18	missing	F	AD	Any substance disorder, Alcohol disorder, Drug diagnosis, Nicotine dependence, Conduct disorder, Oppositional defiant disorder, ADHD, Depression, Any disruptive disorder	Self-report	Self-report	I	36	1020	.19	.13	.25
McGrath, et al., 1999	USA	12–18	49%	B	AD	Academic achievement	Self-report	Self-report, Teacher	I	24	454	.13	.03	.22
Miller, 2001	USA	6–11	57%	M	T	Asthma	Self-report	Mother, Other	Q (parents), Other (children)	36	3938	.08	−.05	.21
Morgenstern, et al., 2013	Germany	12–18	missing	B	T	Tobacco, School performance	Self-report	Self-report	Q	36	1320	.12	.03	.21
Naicker, et al., 2012	Canada	12–18	27%	B	A	Emotional disorder	Self-report	Self-report	Q		937	.10	−.10	.29
Nigg et al., 2006	USA	12–18	missing	MF	AD	ADHD, Response inhibition, Set shifting and working memory, Conduct symptoms, IQ	Self-report	Self-report, Teacher	I	180	498	.06	.00	.13
Pagani, et al., 2016	Canada	6–11	57%	M	T	Being overweight/obese	Self-report	Other	I (parents), Other (children)	36	1323	.14	.00	.27
Power, et al., 2005	USA	12–18	missing	F	A	Alcohol	Self-report	Self-report	Q	6	743	.00	−.20	.20
Roosa, et al., 1990	USA	12–18	missing	B	AD	Anxiety, Depression	Child	Self-report	Q	3	145	.30	.13	.45
Selya, et al., 2012	USA	12–18	57%	MF	T	Tobacco	Self-report, Child	Self-report	Q	42	1092	.20	.06	.33
Shortt, et al., 2007	Australia	12–18	missing	B	A, T	Alcohol	Self-report	Self-report	Q	12	1166	.13	−.01	.26
Thacher et al., 2016	Sweden	12–18	Girls/ boys analysed separately	MFB	T	Allergies	Child, Spouse	Parents	Q	144	3316	.06	−.02	.13
Thompson & Wilsnack, 1987	USA	missing	0%	MF	A	Alcohol	Child	Self-report	Q	48	712	.09	.06	.13
Ting, et al., 2015	Taiwan	12–18	0%	B	A	Alcohol	Self-report	Self-report	Q	48	496	.13	−.02	.27
Tjora, et al., 2011	Norway	12–18	54%	MF	T	Alcohol	Self-report	Self-report	Q	60	1052	.18	.04	.31
Unger et al., 2006	USA	6–11	53%	B	T	Tobacco		Self-report	Q	12	1940	.10	−.10	.29
Van Der Vorst et al., 2013	Canada	12–18	48%	B	A	Alcohol	Child	Self-report	Q	12	1142	.14	−.06	.33
Vella, et al., 2015	Australian	12–18	53%	M	T	Health-related Quality of Life	Self-report	Self-report	I	120	2785	.25	.12	.38
Windham et al., 2004	USA	0–5	29%	M	A, D	Physical abuse, Self-esteem	Self-report	Self-report	I		541	.09	−.02	.19
Wong, et al., 2011	USA	12–18	47%	MF	AD	Suicidal thoughts, Self-harm	Self-report	Self-report	I	96	392	.02	−.10	.15
Xie, et al., 2013	China	12–18	missing	B	T	Tobacco	Child	Self-report	Q		3572	.08	−.03	.19

*Note. N* = average sample size available for analyses; Gender parents: M = mother, F = father, C = child, MF = mother and father separately, MFB = both parents included but not reported on separately; Parental consumption: T = tobacco, A = alcohol, D = drug use, DD = drug use disorder, AD = alcohol use disorder, A = any substance use; Method of assessment: I = interview, Q = Questionnaire.

### Parental substance abuse and child well-being

The three-level random-effects meta-analysis yielded a statistically significant, small association between parental substance abuse and child well-being (*r* = .15), indicating that parental substance abuse was associated with poorer child well-being levels over time. Effect sizes differed significantly both between, 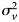 = .003; χ^2^(1) = 17.26, *p* < .001, and within, 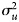
= .009; χ^2^ (1) = 14,804.78, *p* < .001, studies; 21% of the total variance was attributable to differences between studies, and 64% of the total variance was due to differences within studies.

### Moderator analyses

Table 2 presents the results for substantive and methodological moderators. Parental “drug use disorder” (*n* = 2), “any substance abuse” (*n* = 2), “social” (*n* = 1) and “economic” (*n* = 0) child well-being subgroups were excluded from moderator analyses due to an insufficient number of studies. Significant differences were found across parental substance abuse type, with pairwise comparisons revealing that the mean association was significantly stronger for parental drug use compared to alcohol use, *t* (205) = 3.75, *p* < .001, tobacco use, *t* (205) = 3.66, *p* < .001, and alcohol use disorder, *t* (205) = 2.83, *p* = .005. Other pairwise differences were not statistically different. Adding the parental substance abuse type moderator and the child well-being domain moderator explained 8.6% of the within-study variance. We did not find significant effects for the other substantive or methodological moderators. For substances other than tobacco or alcohol, cannabis was specifically identified in 71% of the drug-related category (*k* studies = 5; *n* (effect sizes) = 10). Sensitivity analysis yielded a significant effect of parental cannabis use on child well-being (*r* = .23, 95% CI [.14, .32], *p* < .001).

**Table 2.** Results of the three-level meta-analysis models

	*k* studies	*n*	Effect Size		Test Statistic
			*r*	95% CI	
**Random-effects model**					
Overall effect size	56	220	.15	[.12, .17]	*t*(219) = 12.94, *p <* .001
**Mixed-effects models Substantive Moderators**					
Region					*F*(2, 208) = 1.01, *p* = .365
North America	35	138	.13	[.10, .16]	
Europe	9	46	.16	[.12, .21]	
Australasia	9	27	.17	[.12, .22]	
Age of child					*F*(2, 215) = 0.62, *p* = .541
0 to 5 years	6	22	.18	[.11, .24]	
6 to 11 years	8	25	.14	[.08, .20]	
12 to 18 years	41	171	.14	[.11, .16]	
Gender of child (% female)	41	181			*t*(179) = -0.16, *p* = .873
Gender of parent					*F*(2, 217) = 0.86, *p* = .426
Father	25	82	.16	[.13, .19]	
Mother	16	59	.14	[.10, .18]	
Both	31	79	.13	[.10, .17]	
Parental substance abuse					*F*(3, 205) = 50.15, *p* = .002
Alcohol^a^	21	83	.13	[.09, .16]	
Tobacco^b^	25	70	.13	[.10, .16]	
Drug use^a,b,c,d^	8	14	.25	[.19, .31]	
Alcohol use disorder^c^	11	42	.14	[.09, .19]	
Child well-being dimension					*F*(2, 214) = 20.65, *p* = .073
Physical	38	162	.15	[.12, .17]	
Psychological	16	42	.15	[.11, .19]	
Cognitive	4	13	.06	[-.02, .14]	
**Methodological Moderators**					
Informant on parental substance abuse					*t*(212) = -10.08, *p* = .280
Parent self-report	47	159	.15	[.13, .18]	
Child report	7	55	.12	[.06, .17]	
Informant on child well-being					*F*(2, 203) = 10.53, *p* = .220
Child self-report	42	166	.13	[.11, .16]	
Parent report	12	33	.17	[.12, .22]	
Teacher report	4	7	.10	[.02, .19]	
Shared informant					*t*(218) = 10.34, *p* = .182
Not shared	44	142	.14	[.11, .16]	
Shared	17	78	.17	[.13, .21]	
Mode parental substance abuse					*t*(169) = -0.29, *p* =.770
Interview	24	83	.15	[.12, .18]	
Questionnaire	27	88	.14	[.11, .18]	
Mode child well-being					*t*(174) = 0.02, *p* = .998
Interview	17	57	.14	[.11, .18]	
Questionnaire	26	119	.14	[.11, .17]	
Time lag association (months)	51	195			*t*(193) = -0.43, *p* = .667

*Note.* Some moderators were missing for certain studies. Each study can contribute multiple effect sizes, thus study sample size across subgroups can exceed the total study sample size for the Level 2 moderators. Within each moderator having more than two subgroups, identical letter superscripts indicate significant (*p* < .05) pairwise comparisons between subgroups.

### Risk of bias

Three-level mixed-effect models did not reveal a statistically significant effect on the parental substance abuse–child well-being association for the methodological moderators: power, *t* (218) = 0.97, *p* = .332; retention rate, *t* (159) = 0.06, *p* = .952; sample representativeness, *t* (122) = -1.92, *p* = .058; properties of the parental substance use assessment tool, *t* (212) = 0.11, *p* = .913; properties of the child well-being assessment tool, *F* (2, 215) = 0.17, *p* = .844); or adjustment for confounding, *t* (218) = 0.96, *p* =.336.

**Figure 2.**
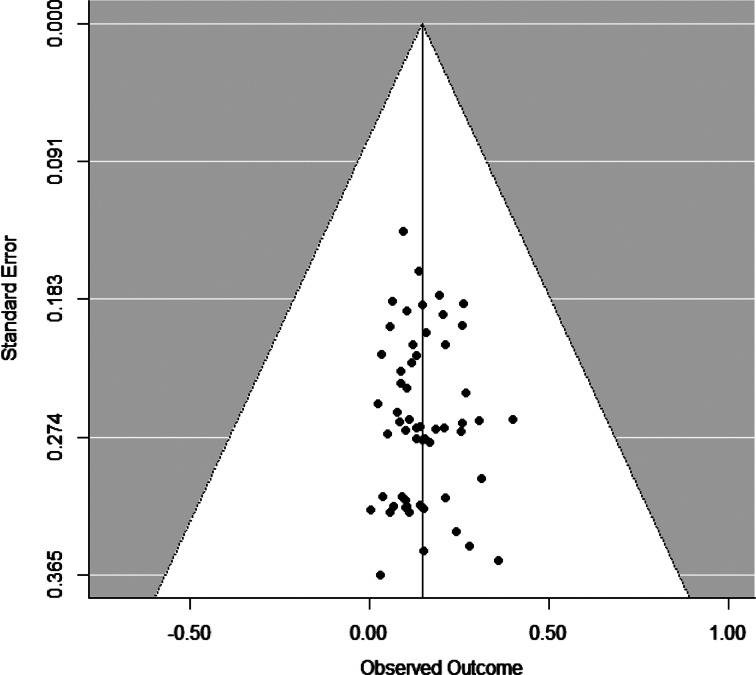
Funnel plot for study-level mean effect sizes.

The funnel plot was funnel-shaped (Figure 2), and Egger's method found no statistically significant asymmetry, *t* (54) = 0.44, *p* = .664, suggesting no indication of publication bias. The sensitivity analyses corroborated these findings. Analyses revealed that the adjusted (*r* = .14) and unadjusted (*r* = .15) population effect size estimates were almost identical under different a priori weight functions. Overall, these analyses indicate that it is highly unlikely that publication bias influenced the meta-analytic results.

## Discussion

We conducted a meta-analysis to examine the association between various indices of parental substance abuse and child well-being over time. A total of 56 studies yielding 220 dependent effect sizes met our inclusion criteria. We found a statistically significant, small longitudinal relationship between parental substance abuse and child well-being, with significant differences both between and within studies. The association between parental substance abuse and child well-being did not differ according to the length of the period between recording the parental substance abuse and the subsequent child well-being outcome, suggesting an enduring effect over time (Evans, Li, & Whipple, 2013). These results indicate that parental substance abuse is a risk factor for subsequent child well-being. The prevalence of substance use and parenthood points to the clinical significance of this risk factor. However, we caution readers to avoid interpreting parental substance abuse as a *causal* risk factor (see Kraemer et al., 1997 for a discussion of risk factor typology) and explicitly recognize that parental substance abuse and child well-being may be caused by the same third variable (e.g., financial or marital problems) or that both concepts can have a reciprocal relationship.

The longitudinal associations observed in the current meta-analysis were heterogeneous, with significant differences relating to the parental substance abuse type. Parental alcohol use (whether dependent or not) had similar secondary effects on children as parental tobacco use, suggesting that recreational alcohol use is as harmful for child well-being as secondary tobacco use and alcohol use disorder. Parental drug use yielded the strongest effect, which could (partially) be due to the illegal nature of these substances that could result in additional disruptive effects on families, such as fines, arrest, or custodial sentences. It should be noted that none of the studies considered prescription drug use, which is an important shortcoming given concerns over prescription opioids (Cicero, Ellis, Surratt, & Kurtz, 2014). Parental substance abuse had similar effects on the physical, psychological, and cognitive well-being of their children. The effects did not differ for fathers, mothers, sons, or daughters, nor according to the age of the child. Although small in magnitude, the effect of parental substance abuse on child well-being has a clear public health significance given the high prevalence of substance abuse (Cumming, 2014).

Our findings corroborate the conjecture that the effect of parental substance abuse on child well-being manifests through the lens of the family environment, with broad detrimental effects on physical, psychological, and cognitive well-being. The substantial unexplained variance that remained in our meta-analysis combined with an effect across substance abuse types could arise from unmeasured factors influencing child well-being that are common across all types of parental substance abuse. If targeting parental substance abuse is to be used in family-centred interventions to improve child outcomes (Calhoun, Conner, Miller, & Messina, 2015), then it will be necessary to further investigate how parental substance abuse affects child outcomes through the parent-child environment. More generally, working with the context of substance abuse, including the family environment in activities is increasingly seen as important in supporting recovery (Friedmann, Hendrickson, Gerstein, & Zhang, 2004; Oser, Knudsen, Staton-Tindall, & Leukefeld, 2009), and this suggests that both child well-being and parental substance abuse can be addressed through targeting aspects of the family environment.

### Strengths and limitations

This study is the first large-scale review to broadly summarize the longitudinal relationship between parental substance abuse and child well-being. We excluded studies that sampled from specific groups whose membership would be expected to be associated with the child well-being outcome. As such, the reported association might be considered conservative, albeit more representative of the general population by doing so. There was no evidence of publication bias or an effect of methodological (rigor) features on the strength of the association, which further underpins the robustness of the findings. The multilevel approach to meta-analysis allowed us to include all relevant effect sizes, to model between- and within-study heterogeneity, and potential moderators that could explain variance at both levels—features that cannot be addressed with a standard meta-analytic approach. Although the multilevel approach is a powerful technique, some moderator analyses may have suffered from low power due to unbalanced groups. In particular, the subgroup effect of parental substance abuse in very young children was slightly elevated, while the subgroup effect for cognitive well-being was less pronounced. The limited number of studies on these topics could have reduced the power of the omnibus test to statistically detect group differences.

Parents with problematic patterns of using an intoxicating substance are likely to show variation in their use patterns over time, including tolerance and consequent increased consumption of substances to achieve the desired effect. They may also go through repeated withdrawal and relapse. At least for chronic use, there is more than simply the presence or the absence of parental substance abuse that potentially affects child well-being—features that this meta-analysis was unable to consider, as this was not reported in the literature reviewed. Furthermore, there was considerable variation in the way parental substance abuse was measured across studies. Some combined substance abuse into a nebulous “any substance” category, some measured frequency, and others reported on recency of use. Given the investment in time and resources required by longitudinal studies, it is important that measurement is given careful consideration in future research and a range of metrics are taken to ensure that parental substance abuse can be appropriately captured. It is feasible that consumption heterogeneity in substances used (e.g., polysubstance abuse, frequency, dose, and physical dependence) would provide additional insights.

Although our results highlight that parental substance abuse is detrimental to children and adolescents, our findings are tempered by the limited number of studies that examined cognitive, social, or economic child well-being outcomes. Only three of the five dimensions of child well-being had sufficient data to be analyzed; therefore, the reported association should be interpreted as a deprecated form of child well-being rather than one that encompasses all aspects of well-being as we intended to quantify.

Within primary studies, well-being outcomes have likely been selected on a like-for-like basis such that parental substance abuse (e.g., tobacco) is expected to have implications for children's health (e.g., respiratory health), and parental alcohol use is expected to have implications for children's subsequent alcohol use, while educational attainment and other aspects of child well-being have been largely ignored. This is notable because the likely mechanisms—that parental substance abuse affects the parent-child relationship–suggests that a broader set of outcomes should be considered and that these gaps in the literature imply that child well-being is not sufficiently investigated to draw firm conclusions about all facets of well-being.

Moreover, as parental substance abuse measures were variously defined from “any substance abuse” to specific substances, there is potential variability in harm by substance type, other than alcohol and tobacco, which we were unable to capture due to a paucity of studies and poorly operationalized measures. Gender differences were also poorly operationalized, with many studies failing to separately capture father and mother differences between boys and girls. Just a third of the included studies considered mothers and fathers separately, only three studies differentiated between male and female children, and only a quarter considered children under 12 years of age. No study supplemented self-report data with objective outcomes, such as data from school or medical records. Therefore, future research needs to improve efforts to account for the multidimensionality of well-being, substance abuse, and features that could moderate effects, as it is possible that the effect of parental substance abuse is conditional on other factors that have not yet been formally documented. The generalizability of the results may also be limited due to a preponderance of studies emerging from North America. These biases and omissions in the research literature offer important opportunities to improve our understanding of the effects of parental substance abuse on child well-being.

### Conclusion

This meta-analysis found that children exposed to parents who consume alcohol (both dependent and non-dependent), tobacco, or other psychoactive drugs experience a detrimental long-term effect on their well-being. However, there is a need for longitudinal studies that more accurately capture the effects of parental psychoactive substance abuse on the full scope of childhood well-being outcomes and integrate substance abuse into models that specify the precise conditions under which parental behavior determines child well-being. These results can inform the development of family-oriented initiatives directed at improving child well-being that may also assist parental recovery.

## Supplementary material

For supplementary material accompanying this paper visit https://doi.org/10.1017/S0954579419000749. click here to view supplementary material
