# Supplementary material for: The Enduring Effects of Parental Alcohol, Tobacco, and Drug Use on Child Well-being: A Multilevel Meta-Analysis
Source: Dev Psychopathol. 2020 May;32(2):765–78. doi: 10.1017/S0954579419000749 (PMC7525110; doi:10.1017/S0954579419000749)
Supplement: Supplementary file 1 [file S0954579419000749sup001.docx]

The Enduring Effects of Parental Alcohol, Tobacco, and Drug Use on Child Well-being:

A Multi-Level Meta-analysis

**Supplement**

**Contents**

**Supplement 1** – Methods and search terms used to identify studies included in the meta-analysis

**Supplement 1**

To map outcomes to the five dimensions of wellbeing and parental consumption types an initial scoping study was completed and an exhaustive list of terms derived.

**Key concept 1 - Parental consumption behaviour**

Alcohol Consumption; Alcohol Drinking; Alcohol Use; Drinking behaviour; Binge drinking; Alcohol Abuse; Alcohol Addiction; Alcohol Dependence; Alcohol Disorder; Alcoholism; Alcoholic; Tobacco Use; Tobacco Consumption; Tobacco Smoking; Cigarette Smoking; Tobacco Use Disorder; Tobacco Dependence; Nicotine Use Disorder; Nicotine Dependence; Drug Use; Drug Abuse; Drug Addiction; Drug Dependence; Drug Disorder; Drug Use Disorder; Substance Use; Substance Abuse; Substance Dependence; Substance Addiction; Substance Disorder

**Key concept 2 - Child well-being (cognitive)**

Academic incompetence; Concentration; Developmental delay; School behavior problems; Academic achievement; Cognitive ability; Quality of school life; School function; School integration; School-related behaviors; Self-concept of academic ability

**Key concept 3 - Child well-being (economic)**

Child support

**Key concept 4 - Child well-being (physical)**

Health compromising behaviors; Physical abuse; Physical manifestations of stress; Physical symptoms of illness; Substance abuse; Substance use; Exercise; Full immunization coverage; Health promotion behavior; Nutrition; Personal body care; Physical appearance; Physical health; Safety-related behavior

**Key concept 5 - Child well-being (psychological)**

Adjustment problems; Aggression; Anger; Anxiety; Behaviour problems; Delinquency; Depression; Despondency; Deviant behaviours; Distress; Eating-related behaviour problems ; Emotional problems; Externalizing behaviours; Fearfulness; Fears of future; General psychological distress; Health compromising behaviours; Hopelessness; Hyperactivity; Impaired concentration; Inattention; Internalizing behaviours; Irritability; Loneliness; Maladjustment; Negative affect; Nervousness; Neuroticism; Nightmares; Non-psychiatric disturbances; Panic; Psychiatric symptoms; Psychological distress; Psychological health symptoms; Recurrent memories of bullying; Self-centeredness; Self-inflicted injury; Spitefulness; Stress; Suicidal ideation/attempts; Weeping; Whining; Withdrawal; Adjustment; Attachment; Autonomy; Behavioural competence; Behavioural functioning; Capacity to love; Cheerfulness; Competence; Coping; Emotional adjustment; Emotional support; Expansiveness; Expectancy for success; Fulfilment; Global satisfaction; Global self-worth; Happiness; Hopefulness; Initiative; Life satisfaction; Mastery; Mental health; Overall functioning; Positive affect; Positive attitude toward school; Positive attitude toward self; Positive mood; Purpose in life; Resilience; Satisfaction with gender; Satisfaction with self; Self-concept; Self-esteem; Self-identified strengths; Self-reliance; Self-worth; Socio-emotional adjustment; Stress management

**Key concept 6 - Child well-being (social)**

Anti-social behavior; Negative life events; Peer problems; Poverty; Troubled home relationships; Family relations; Parent-child relations; Participation in cultural activities; Prosocial behaviors; Prosocial values; Quality of life; Relationships in the home; Relationships in the school; Relationships with peers; Social acceptance; Social skills; Social support; Socioeconomic status.

These formed the basis of the search terms, with an expectation that the search would be over inclusive. Below are the search strings used in PubMed, Embase, Medline and PsychInfo:

**PubMed**

(parent* OR mother* OR mum* OR father* OR dad*) AND (“Alcohol Consumption” OR “Alcohol Drinking” OR “Alcohol Use” OR “Drinking behaviour” OR “Binge drinking” OR “Alcohol Abuse” OR “Alcohol Addiction” OR “Alcohol Dependence” OR “Alcohol Disorder” OR Alcoholism OR Alcoholic* OR “Tobacco Use” OR “Tobacco Consumption” OR “Tobacco Smoking” OR “Cigarette Smoking” OR “Tobacco Use Disorder” OR “Tobacco Dependence” OR “Nicotine Use Disorder” OR “Nicotine Dependence” OR “Drug Use” OR “Drug Abuse” OR “Drug Addiction” OR “Drug Dependence” OR “Drug Disorder” OR “Drug Use Disorder” OR “Substance Use” OR “Substance Abuse” OR “Substance Dependence” OR “Substance Addiction” OR “Substance Disorder”) AND (child* OR adoles* OR offspring OR teen*) AND (Learning OR “Cognitive functioning” OR “Academic incompetence” OR Concentration OR “Developmental delay” OR “School behavior problems” OR “Academic achievement” OR “Cognitive ability” OR “Quality of school life” OR “School function” OR “School integration” OR “School-related behaviors” OR “Self-concept of academic ability” OR “child support” OR “health costs“ OR “Health status” OR “Child nutrition disorder” OR “Nutrition Disorder” OR “Parenteral Nutrition” OR “Infant Nutrition Disorder” OR Exercise OR “Physical abuse” OR “Physical manifestations of stress” OR “Physical symptoms of illness” OR “Full immunization coverage” OR Nutrition OR “Personal body care” OR “Physical appearance” OR “Physical health” OR “Safety-related behaviour” OR Well-being OR “Mental health” OR “Psychological Disorder” OR “Mental Disorder” OR “Adjustment problems” OR Aggression OR Anger OR Anxiety OR “Behaviour problems” OR Delinquency OR Depression OR Despondency OR “Deviant behaviours” OR Distress OR “Eating-related behaviour problems” OR “Emotional problems” OR “Externalizing behaviours” OR Fearfulness OR “Fears of future” OR “General psychological distress” OR “Health compromising behaviours” OR Hopelessness OR Hyperactivity OR “Impaired concentration” OR Inattention OR “Internalizing behaviours” OR Irritability OR Loneliness OR Maladjustment OR “Negative affect” OR Nervousness OR Neuroticism OR Nightmares OR “Non-psychiatric disturbances” OR Panic OR “Psychiatric symptoms” OR “Psychological distress” OR “Psychological health symptoms” OR “Recurrent memories of bullying” OR “Self-centeredness” OR “Self-inflicted injury” OR Spitefulness OR Stress OR “Suicidal ideation” OR Weeping OR Whining OR Withdrawal OR Adjustment OR Attachment OR Autonomy OR “Behavioural competence” OR “Behavioural functioning” OR “Capacity to love” OR Cheerfulness OR Competence OR Coping OR “Emotional adjustment” OR “Emotional support” OR “Expectancy for success” OR Fulfilment OR “Global satisfaction” OR “Global self-worth” OR Happiness OR Hopefulness OR Initiative OR “Life satisfaction” OR Mastery OR “Mental health” OR “Overall functioning” OR “Positive affect” OR “Positive attitude toward school” OR “Positive attitude towards self” OR “Positive mood” OR “Purpose in life” OR Resilience OR “Satisfaction with gender” OR “Satisfaction with self” OR Self-concept OR “Social behaviour” OR “quality of life” OR “Anti-social behaviour” OR “Negative life events” OR “Peer problems” OR Poverty OR “Troubled home relationships” OR “Family relations” OR “Parent-child relations” OR “Participation in cultural activities” OR “Prosocial behaviors” OR “Prosocial values” OR “Relationships in the home” OR “Relationships in the school” OR “Relationships with peers” OR “Social acceptance” OR “Social skills” OR “Social support” OR “Socioeconomic status”) AND (longitudinal OR cohort OR panel OR cross-sectional)

Ovid (includes Embase, Medline and PsychInfo)

1. exp parents/

2. parent*.tw.

3. mother*.tw.

4. mum*.tw.

5. father*.tw.

6. dad*.tw.

7. drinking behavior/ or alcohol drinking/ or binge drinking/

8. Alcoholism/

9. (alcohol adj1 abuse).tw.

10. (alcohol adj1 addiction).tw.

11. (alcohol adj1 (dependenc* or disorder*)).tw.

12. alcoholism.tw.

13. alcoholic*.tw.

14. (binge adj1 drink*).tw.

15. (drinking adj1 behavior*).tw.

16. (alcohol adj1 consumption).tw.

17. (alcohol adj1 "use").tw.

18. "tobacco use"/ or smoking/

19. "Tobacco Use Disorder"/

20. Smoking/

21. (tobacco adj1 "use").tw.

22. (tobacco adj1 dependenc*).tw.

23. (tobacco adj1 consumption).tw.

24. (tobacco adj1 smoking).tw.

25. (cigarette adj1 smoking).tw.

26. (tobacco adj1 dependenc*).tw.

27. (tobacco adj1 "use" adj1 disorder).tw.

28. substance abuse/ or drug abuse/

29. (drug adj1 "use").tw.

30. (drug adj1 abuse).tw.

31. (drug adj1 addiction).tw.

32. (drug adj1 dependenc*).tw.

33. (drug adj1 "use" adj1 disorder).tw.

34. (substance adj1 "use").tw.

35. (substance adj1 dependenc*).tw.

36. (substance adj1 addiction).tw.

37. (substance adj1 disorder).tw.

38. adolescent/ or child/

39. child*.tw.

40. teen*.tw.

41. adolescent*.tw.

42. learning/ or cognitive functioning/

43. (academic adj1 incompetence).tw.

44. concentration.tw.

45. (development adj1 delay).tw.

46. (school adj1 behavior adj1 problems).tw.

47. (academic adj1 achievement).tw.

48. (cognitive adj1 ability).tw.

49. (quality adj1 of adj1 school adj1 life).tw.

50. (school adj1 function).tw.

51. (school adj1 integration).tw.

52. (school-related adj1 behavior*).tw.

53. (self-concept adj1 of adj1 academic adj1 ability).tw.

54. 1 or 2 or 3 or 4 or 5 or 6

55. 7 or 8 or 9 or 10 or 11 or 12 or 13 or 14 or 15 or 16 or 17 or 18 or 19 or 20 or 21 or 22 or 23 or 24 or 25 or 26 or 27 or 28 or 29 or 30 or 31 or 32 or 33 or 34 or 35 or 36 or 37

56. 38 or 39 or 40 or 41

57. 42 or 43 or 44 or 45 or 46 or 47 or 48 or 49 or 50 or 51 or 52 or 53

58. 54 and 55 and 56 and 57

59. limit 58 to english language

60. Health Status/

61. Child Nutrition Disorders/ or Nutrition Disorders/ or Parenteral Nutrition/ or Infant Nutrition Disorders/

62. Exercise/

63. (physical adj1 abuse).tw.

64. (physical adj1 manifestation adj1 stress).tw.

65. (physical adj1 symptoms adj1 of adj1 illness).tw.

66. immunization.tw.

67. (body adj1 care).tw.

68. (physical adj1 appearance).tw.

69. well-being.tw.

70. (safety-related adj1 behavior).tw.

71. 60 or 61 or 62 or 63 or 64 or 65 or 66 or 67 or 68 or 69 or 70

72. 54 and 55 and 56 and 71

73. limit 72 to english language

74. Economics, Pharmaceutical/ or Economics, Behavioral/ or Economics, Medical/ or Economics/ or Economics, Hospital/ or Economics, Dental/ or Economics, Nursing/

75. (child adj1 support).tw.

76. 74 or 75

77. 54 and 55 and 56 and 76

78. limit 77 to english language

79. social behavior/ or quality of life/

80. (anti-social adj1 behavior).tw.

81. (negative adj1 life adj1 events).tw.

82. (peer adj1 problems).tw.

83. poverty.tw.

84. (troubled adj1 home adj1 relationships).tw.

85. (family adj1 relations).tw.

86. (parent-child adj1 relations).tw.

87. (participation adj1 in adj1 cultural adj1 activities).tw.

88. (prosocial adj1 behavior).tw.

89. (prosocial adj1 values).tw.

90. (quality adj1 of adj1 life).tw.

91. (relationships adj1 in adj1 the adj1 home).tw.

92. (relationships adj1 in adj1 the adj1 school).tw.

93. (relationships adj1 with adj1 peers).tw.

94. (social adj1 skills).tw.

95. (social adj1 support).tw.

96. (socioeconomic adj1 status).tw.

97. 79 or 80 or 81 or 82 or 83 or 84 or 85 or 86 or 87 or 88 or 89 or 90 or 91 or 92 or 93 or 94 or 95 or 96

98. 54 and 55 and 56 and 97

99. limit 98 to english language

100. Depression/ or Mental Health/ or Stress, Psychological/ or Mental Disorders/ or "Quality of Life

101. (adjustment adj1 problems).tw.

102. aggression.tw.

103. anger.tw.

104. anxiety.tw.

105. (behaviour adj1 problem*).tw.

106. deliquency.tw.

107. depression.tw.

108. despondency.tw.

109. (deviant adj1 behaviours).tw.

110. distress.tw.

111. (eating-related adj1 behaviour adj1 problems).tw.

112. (emotional adj1 problems).tw.

113. (externalizing adj1 behaviour*).tw.

114. fearfulness.tw.

115. (fears adj1 of adj1 future).tw.

116. (psychological adj1 distress).tw.

117. (health adj1 compromising adj1 behavior*).tw.

118. hopelessness.tw.

119. hyperactivity.tw.

120. (impaired adj1 concentration).tw.

121. inattention.tw.

122. (internalizing adj1 behaviour*).tw.

123. irritability.tw.

124. loneliness.tw.

125. maladjustment.tw.

126. (negative adj1 affect).tw.

127. nervousness.tw.

128. neuroticism.tw.

129. nightmares.tw.

130. (non-psychiatric adj1 disturbances).tw.

131. panic.tw.

132. (psychiatric adj1 symptom*).tw.

133. (psychological adj1 distress).tw.

134. (psychological adj1 health adj1 symptoms).tw.

135. (memories adj1 of adj1 bullying).tw.

136. self-centeredness.tw.

137. (self-inflicted adj1 injury).tw.

138. spitefulness.tw.

139. stress.tw.

140. (suicidal adj1 ideation).tw.

141. (suicidal adj1 attempts).tw.

142. weeping.tw.

143. whining.tw.

144. withdrawal.tw.

145. adjustment.tw.

146. attachment.tw.

147. autonomy.tw.

148. (behavioral adj1 competence).tw.

149. (behavioral adj1 functioning).tw.

150. (capacity adj1 to adj1 love).tw.

151. cheerfulness.tw.

152. competence.tw.

153. coping.tw.

154. (emotional adj1 adjustment).tw.

155. expansiveness.tw.

156. (expectancy adj1 for adj1 success).tw.

157. fulfilment.tw.

158. (global adj1 satisfaction).tw.

159. (gloabl adj1 self-worth).tw.

160. happiness.tw.

161. hopefulness.tw.

162. initiative.tw.

163. (life adj1 satisfaction).tw.

164. mastery.tw.

165. (mental adj1 health).tw.

166. (overall adj1 functioning).tw.

167. (positive adj1 affect).tw.

168. (positive adj1 attitude adj1 toward adj1 school).tw.

169. (positive adj1 attitude adj1 toward adj1 self).tw.

170. (positive adj1 mood).tw.

171. (purpose adj1 in adj1 life).tw.

172. resilience.tw.

173. (satisfaction adj1 with adj1 gender).tw.

174. (satisfation adj1 with adj1 self).tw.

175. self-concept.tw.

176. self-esteem.tw.

177. (self-identified adj1 strenghts).tw.

178. self-reliance.tw.

179. self-worth.tw.

180. (socio-emotional adj1 adjustment).tw.

181. (stress adj1 management).tw.

182. 100 or 101 or 102 or 103 or 104 or 105 or 106 or 107 or 108 or 109 or 110 or 111 or 112 or 113 or 114 or 115 or 116 or 117 or 118 or 119 or 120 or 121 or 122 or 123 or 124 or 125 or 126 or 127 or 128 or 129 or 130 or 131 or 132 or 133 or 134 or 135 or 136 or 137 or 138 or 139 or 140 or 141 or 142 or 143 or 144 or 145 or 146 or 147 or 148 or 149 or 150 or 151 or 152 or 153 or 154 or 155 or 156 or 157 or 158 or 159 or 160 or 161 or 162 or 163 or 164 or 165 or 166 or 167 or 168 or 169 or 170 or 171 or 172 or 173 or 174 or 175 or 176 or 177 or 178 or 179 or 180 or 181

183. 54 and 55 and 56 and 182

184. limit 183 to english language

185. limit 59 to human

186. limit 73 to human

187. limit 99 to human

188. limit 184 to human

189. longitudinal.tw.

190. cohort.tw.

191. panel.tw.

192. cross-sectional.tw.

193. 189 or 190 or 191 or 192

194. limit 78 to human

195. 185 or 186 or 187 or 188 or 194

196. 54 and 55 and 56 and 193 and 195

197. 54 and 55 and 56 and 195
